# Characterizing dysregulations via cell-cell communications in Alzheimer’s brains using single-cell transcriptomes

**DOI:** 10.1186/s12868-024-00867-y

**Published:** 2024-05-13

**Authors:** Che Yu Lee, Dylan Riffle, Yifeng Xiong, Nadia Momtaz, Yutong Lei, Joseph M. Pariser, Diptanshu Sikdar, Ahyeon Hwang, Ziheng Duan, Jing Zhang

**Affiliations:** 1 Department of Computer Science, University of California, Irvine, CA USA; 2 Department of Biomedical Engineering, Johns Hopkins University, Baltimore, MD USA; 3 Mathematical, Computational and Systems Biology, University of California, Irvine, CA USA

**Keywords:** Cell-cell communication, Ligand-receptor, Single-nucleus RNA-seq, Brain, Alzheimer’s Disease, Network

## Abstract

**Background:**

Alzheimer’s disease (AD) is a devastating neurodegenerative disorder affecting 44 million people worldwide, leading to cognitive decline, memory loss, and significant impairment in daily functioning. The recent single-cell sequencing technology has revolutionized genetic and genomic resolution by enabling scientists to explore the diversity of gene expression patterns at the finest resolution. Most existing studies have solely focused on molecular perturbations within each cell, but cells live in microenvironments rather than in isolated entities. Here, we leveraged the large-scale and publicly available single-nucleus RNA sequencing in the human prefrontal cortex to investigate cell-to-cell communication in healthy brains and their perturbations in AD. We uniformly processed the snRNA-seq with strict QCs and labeled canonical cell types consistent with the definitions from the BRAIN Initiative Cell Census Network. From ligand and receptor gene expression, we built a high-confidence cell-to-cell communication network to investigate signaling differences between AD and healthy brains.

**Results:**

Specifically, we first performed broad communication pattern analyses to highlight that biologically related cell types in normal brains rely on largely overlapping signaling networks and that the AD brain exhibits the irregular inter-mixing of cell types and signaling pathways. Secondly, we performed a more focused cell-type-centric analysis and found that excitatory neurons in AD have significantly increased their communications to inhibitory neurons, while inhibitory neurons and other non-neuronal cells globally decreased theirs to all cells. Then, we delved deeper with a signaling-centric view, showing that canonical signaling pathways CSF, TGFβ, and CX3C are significantly dysregulated in their signaling to the cell type microglia/PVM and from endothelial to neuronal cells for the WNT pathway. Finally, after extracting 23 known AD risk genes, our intracellular communication analysis revealed a strong connection of extracellular ligand genes APP, APOE, and PSEN1 to intracellular AD risk genes TREM2, ABCA1, and APP in the communication from astrocytes and microglia to neurons.

**Conclusions:**

In summary, with the novel advances in single-cell sequencing technologies, we show that cellular signaling is regulated in a cell-type-specific manner and that improper regulation of extracellular signaling genes is linked to intracellular risk genes, giving the mechanistic intra- and inter-cellular picture of AD.

**Supplementary Information:**

The online version contains supplementary material available at 10.1186/s12868-024-00867-y.

## Background

Alzheimer’s disease is a devastating neurodegenerative disorder that affects 44 million people worldwide, leading to cognitive decline, memory loss, and significant impairment in daily functioning [1–3]. Understanding and studying Alzheimer’s disease is of utmost importance due to its widespread impact on individuals, families, and society as a whole [4–6]. Despite decades of efforts to narrow down several risk genes, the genetic and molecular mechanisms underlying AD are largely unknown. Bulk-tissue sequencing masks the heterogeneity of gene expression underlying distinct cell types [7]. As a result, we still face significant hurdles in developing effective treatment or prevention for this devastating disease.

The recent single-cell sequencing technology has revolutionized genetic and genomic studies by simultaneously profiling molecular signatures across thousands to millions of cells. It enables scientists to explore cellular diversity, gene expression patterns, and cellular interactions in complex tissues and health conditions. It has allowed us to identify unique cell types, discover disease-specific cellular signatures, and unravel the intricate mechanisms underlying genetic disorders [8–13]. As a result, several single-cell genomic research studies have been conducted to investigate the disease pathology and provide new molecular insights in AD research. For instance, Mathys et al. performed population-scale single-nucleus RNA sequencing (snRNA-seq) in post-mortem human prefrontal cortices from AD patients and healthy controls to reveal both cell-type-specific and cell-type-shared transcription perturbation signatures in AD [14]. On the other hand, Morabito et al. performed single-cell epigenetic and transcriptomic profiling and identified cell-type-specific cis-regulatory elements (CREs) and transcription factors (TF) that may mediate gene-regulatory changes in the late-stage AD [15]. Most of the existing studies have solely focused on molecular perturbations within each cell. However, cells are not isolated entities but live in a microenvironment, or cell niche, composed of dynamically interacting entities, including extracellular matrix, neighboring cells, and soluble factors [8, 16, 17]. The complex and multidirectional interplay between these factors (and their properties) plays crucial roles in tissue development, cellular responses, disease progression, and therapeutic interventions [18–20]. Understanding and manipulating this relationship can provide insights into disease mechanisms and guide the development of novel therapeutic strategies.

To fill this gap of multi-cellular interplay, we leveraged the large-scale and publicly available single-nucleus RNA sequencing (snRNA-seq) in the human prefrontal cortex (PFC) to investigate cell-to-cell communication (C2C) patterns and their perturbations in AD patients. We first downloaded 48 snRNA-seq samples (24 AD and 24 control) and uniformly re-processed them with strict QCs. Next, we identified canonical cell types and their subclasses, consistent with cell type definitions from BRAIN Initiative Cell Census Network (BICCN) [21]. With such uniformly processed data, we built a high-confidence cell-to-cell communication network composed of signaling genes and inferred the major signaling pathway patterns in AD and healthy brains separately. Interestingly, we found that healthy brains form clear C2C patterns with distinct signaling usage, which has been significantly disrupted in AD brains. When compared to control, Alzheimer’s excitatory cell types seem to be sending more communication signals specifically to the inhibitory cell types, while inhibitory and non-neuronal cell types globally decreased their outgoing signals to most cell types. We then delved deeper with a signaling-centric view. We found that many previously reported signaling pathways, such as CSF, TGFβ, and CX3C, are significantly dysregulated in their signaling to the cell type microglia/PVM [22–24]. In contrast, the AD-relevant WNT pathway is dysregulated in its signaling from endothelial to neuronal cells in AD [25]. Finally, we calculated the regulatory scores of ligand genes and discovered, specifically, a strong connection of extracellular ligand genes APP, APOE, and PSEN1 to intracellular AD risk genes TREM2, ABCA1, and APP in the communication from astrocytes and microglia to neurons. In summary, with the novel advances in single-cell sequencing technologies, we show that cellular signaling is regulated in a cell-type-specific manner and that improper regulation of extracellular signaling genes is linked to intracellular risk genes, garnering cross-cell-type mechanistic insights behind Alzheimer’s Disease.

## Methods

### snRNA-seq processing

We first mapped the raw reads and generated a cell-by-count matrix using CellRanger count v6.0 [26]. Next, to more carefully separate out true cells from empty droplets with ambient RNA, we used the program remove-background from the CellBender package [27]. After filtering cells based on the lower bounds, we removed 1,135 genes included in the MitoCarta v3.0 database [28] such as mitochondrial genes and certain genes highly correlated with RNA sample quality [29]. Next, doublets were identified using a combination of two computational methods Scrublet [30] and DoubletDetection [31]. The intersection of high-quality cells was taken from both software. Furthermore, cells with more than 10% mitochondrial gene expression, fewer than 200 genes, or fewer than 500 UMIs were excluded from downstream analysis. After these removals, we aggregated the demultiplexed samples again in Pegasus [32] for robust gene identification, highly variable gene selection (5,000 genes chosen), principal component analysis (PCA), batch correction using Harmony [33], nearest-neighbor detection, Leiden clustering, and Uniform Manifold Approximation and Projection (UMAP) dimensionality reduction.

### Cell type annotation

**Fig. 1:**
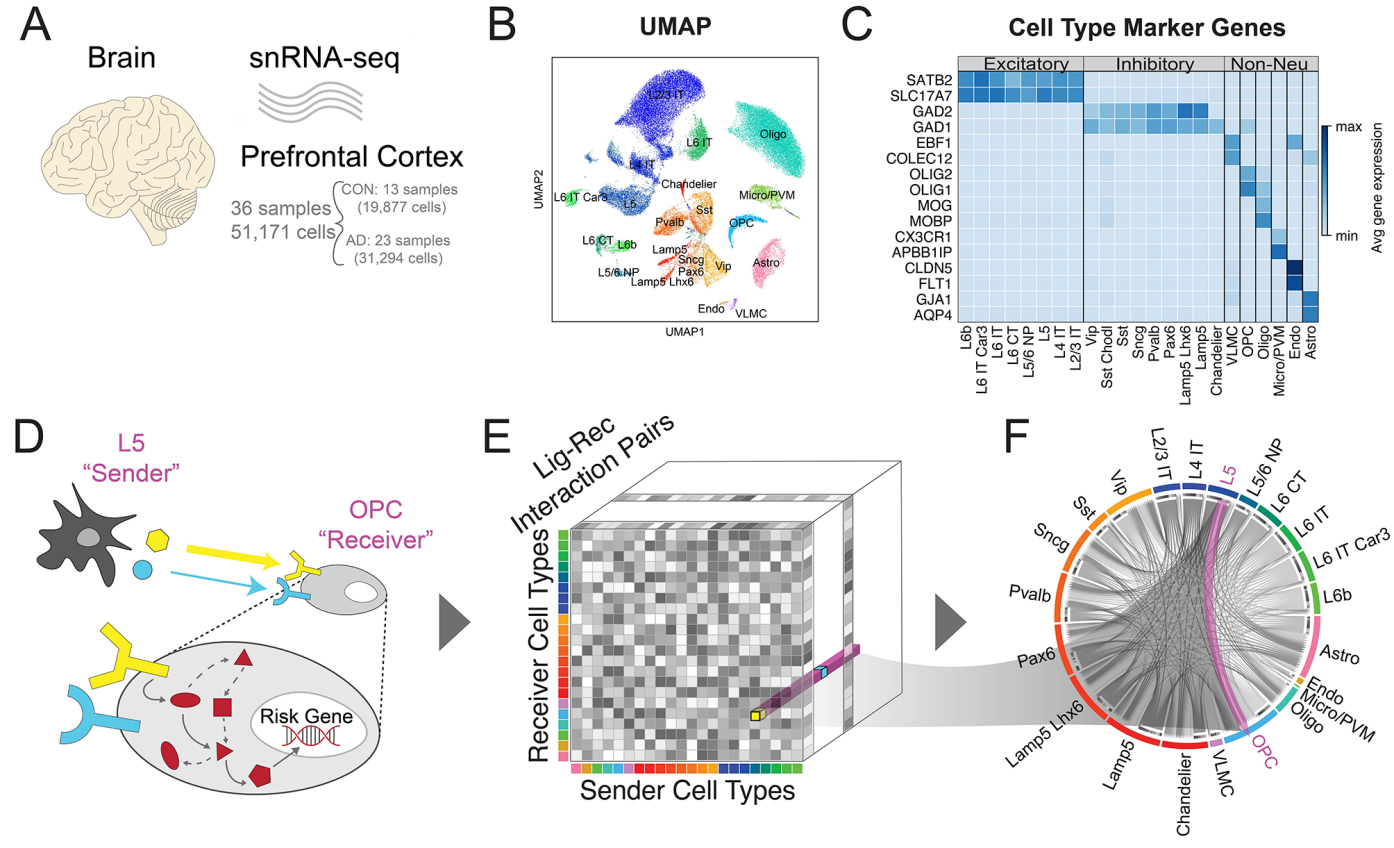
Data overview. (**A**) Schematic of the Accelerating Medicines Partnership Program for Alzheimer’s Disease Consortium Data. (**B**) UMAP-embedding of the single-cell data labeled by cell type. (**C**) Table of marker genes for each cell type. (**D**) Schematic of the cell-to-cell communication analysis performed in the paper. Example of one communication (i.e. L5 to OPC) is highlighted. (**E**) The data structure of our cell-to-cell communication analysis. It is a three-dimensional matrix representing the communication strength between any sender and receiver cell type pair via a specific ligand-receptor pair. The L5 to OPC is an example array in our communication matrix. (**F**) An overall cell-to-cell communication network, with an example that highlights the signaling from L5 neuron to OPC glial cell

We used Pegasus’ *infer_cell_types* function to associate the Leiden clusters with reference cell types based on the hybrid marker gene sets obtained from merging BICCN’s neuronal subclass markers and Ma et al’s non-neuronal subclasses [34]. The broad cell types’ and non-neuronal marker genes can be found in Fig. 1, while the sub-cell-types’ marker genes for the neuronal cells in Fig. S1. The final subclass annotations included the following (Table 1):

**Table 1:** Groupings of each cell type

Cell Type	Cell Subtypes
Excitatory Neurons	L2/3 IT, L4 IT, L5, L5/6 NP, L6 CT, L6 IT, L6 IT Car3, L6b
Inhibitory Neurons	Chandelier, Lamp5, Lamp5 Lhx6, Pax6, Pvalb, Sncg, Sst, Sst Chodl, Vip
Non-Neuronal Cells	Astro, Endo, VLMC, Micro/PVM, Oligo, OPC

### Intercellular communication analyses

#### Individual cell-to-cell communication network

Here, we applied the standard workflow of CellChat (v1.5.0) on single-cell gene expression of ligands and receptors [35]. Based on an existing database of ligand-receptor pairs, we utilized the default parameters ‘mean = trimean’ and ‘trim = 0.1’ to infer a cell-to-cell communication network. Two separate analyses were done for each condition in the AD dataset, namely control and Alzheimer’s.

#### Spatial validation of control cell-to-cell network

We utilized processed, deconvoluted spatial cell data (Sample Name “Br6432_post”) generated by 10X Visium technology to perform a limited amount of validation [36]. After showing the layer-specificity of specific cell types (Fig. S2), a spatially-aware cell-to-cell communication matrix was generated with ‘mean = trimean’ and ‘trim = 0.1’. Pearson, Kendall, and Spearman correlations between the spatially aware and the non-spatially aware communication matrices were calculated.

#### Differential cell-to-cell communication network

Next, we moved from individual cell-to-cell communication analyses to differential analyses. We began our analyses by first normalizing the 3-dimensional matrices (Sender cell types x Receiver cell types x Lig-Rec interactions) in each of the CellChat objects to correct for batch effects in snRNA-seq, this time specifically for signaling genes. Firstly, for pattern analysis, we utilized the Brunet algorithm with ‘seeding = random’, ‘number of runs = 200’, and ‘rank = 3’ [37]. The NMF signaling pattern analysis works by decomposing the communication matrix into two smaller sub-matrices to find the underlying pattern among the cell types and their corresponding signaling pathways. Secondly, for our cell-type-centric analyses, an element-wise subtraction of the two cell-to-cell networks was performed. Values per cell type were summed to inform cell-type-centric input/output analyses. Finally, for our signaling-centric analyses, CellChat’s “rankNet” function performed a paired sample Wilcoxon test comparing all possible sender-receiver cell type pairs between AD and CON groups. A significant *P*-value indicates that all interactions from one diagnosis consistently rank lower than those from the other. A *P*-value of 0.05 and a ratio difference of less than 0.95 or more than 1.05 were used to determine statistical significance, following the default parameters benchmarked by the original authors of CellChat.

### AD risk gene extraction

To identify AD risk genes for our research, we intersected the results from a Genome-Wide Association Studies (GWAS) [38, 39], a Whole Exome Sequencing (WES) study [40], and a network-based study to obtain a total of 23 AD risk genes [41]. We obtained 87 risk genes from the GWAS & the exome study and 430 genes from the network-based study. We decided to include the network-based genes, in addition to the GWAS and Exome single-gene analyses, because genes do not act in isolation but in concert with other genes [41]. The intersection of the 23 AD risk genes can be visualized in the Venn diagram (Fig. S3).

### Intracellular communication analyses

We calculated the ligand-gene regulatory scores using NicheNet (v1.1.1) [42]. NicheNet inputs were as follows: sender cells—astrocytes or microglia/PVM; receiver cells—all the neuronal cell types. The extraction of target risk gene input is described above (Methods 2.4). The following filters were used in the NicheNet analysis: ‘n_ligands = 20’, ‘n_targets = 400’, ‘cutoff = 0.25’. We then selected the top 10 ligands and 15 target genes to highlight in our C2C analysis.

## Results

We utilized the publicly available data from the Accelerating Medicines Partnership Program for Alzheimer’s Disease Consortium [14] of 24 AD and 24 health control samples. We uniformly processed the raw fastq files and kept 51,171 nuclei after strict QC (details in Methods 2.1), which included 31,294 nuclei from 23 AD samples and 19,877 nuclei from 13 healthy controls (Fig. 1A). These high-quality nuclei formed eight major cell types characterized by canonical marker genes (Fig. 1B-C). Since both excitatory and inhibitory neurons are composed of heterogeneous subclasses, we further sub-clustered the neuronal cell types by leveraging the existing BICCN reference dataset for PFC, resulting in eight excitatory and nine inhibitory neuron subclasses (details in Methods 2.2 and Fig. S1).

We performed a comprehensive communication analysis, looking at both external intercellular (via CellChat) and internal intracellular communication (via NicheNet). Specifically, we utilized the gene expression patterns of known ligand-receptor pairs from the snRNA-seq data to infer the C2C networks via the popular software package CellChat and connected them with downstream risk genes via NicheNet (Fig. 1D) [35, 42]. We constructed a three-dimensional matrix representing the communication strength between any sender and receiver cell type pair via a specific ligand-receptor interaction (Fig. 1E and Methods 2.3 A). To validate our network, we utilized a neurotypical (aka non-Alzheimer’s) spatial transcriptomics dataset to generate a spatially-aware cell-to-cell network for our control communication signaling [36] (details in Methods 2.3B). The positive correlations validate the correspondence between the two communication networks generated by two independent datasets (Table 2). As a result, this allowed us to confidently aggregate the C2C communication patterns in AD and healthy controls, measure C2C changes between conditions, infer disease-driving signal pathways, and connect risk genes to upstream ligand regulators in a cell-type-specific manner (Fig. 1F). We will discuss the detailed results in the following sections.

**Table 2:** Correlations between the spatial cell-to-cell network and the original cell-to-cell network

	Coefficients	*P*-value
Pearson	0.3079	5.8e-4
Kendall	0.3213	2.7e-7
Spearman	0.4607	1.0e-7

### Communication pattern analysis reveals inter-mixing of cell types and signaling pathways in AD brains

**Fig. 2:**
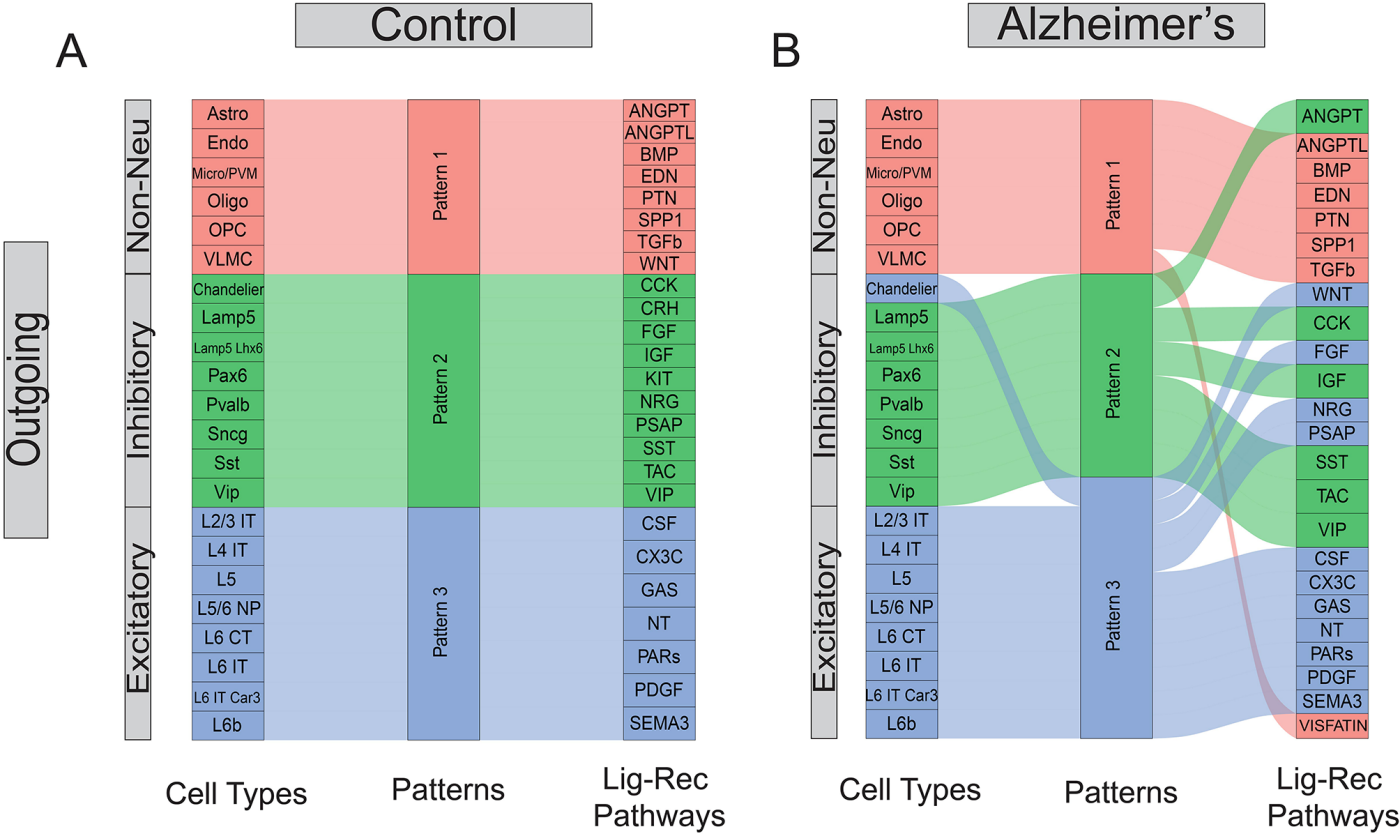
Pattern analysis of cell-to-cell communication. (**A**) Pattern analysis of the outgoing network for the control cells. (**B**) Pattern analysis of the outgoing network for the Alzheimer’s cells. The first column represents the cell types, while the last column represents the ligand-receptor pathways. Each pathway can contain multiple ligand-receptor pairs

With the 3D C2C matrix constructed, we first explored how multiple cell types coordinate intercellular communications using certain pathways in an unsupervised manner. To achieve this goal, we flattened the 3D communication matrix into a 2D sender-by-LigandReceptorPair matrix and performed non-negative matrix factorization (NMF) to identify latent communication groups and their key ligand-receptor signaling contributors [35]. We demonstrated our outgoing C2C network results in the alluvial plot in Fig. 2, where the middle bar represents the latent patterns, and the flow indicates how different signaling pathways (or cell types) belong to each pattern. Interestingly, we found normal brains employ three distinct outgoing communication latent patterns in three major cell groups: excitatory neurons, inhibitory neurons, and non-neuronal cells (Fig. 2A). All of the outgoing non-neuronal cells are characterized by pattern 1, dominated by biologically relevant pathways named after genes such as ANGPT, BMP, SPP1, and TGFβ [43–45]. Inhibitory neurons are represented by pattern 2, driven by expected signaling pathways such as VIP, SST, CCK, and CRH [46–49] while excitatory neurons are characterized by pattern 3, driven by signaling pathways such as CSF, SEMA3, and NT [50–52]. These results show that biologically-related cell types in normal brains rely on largely overlapping signaling networks.

In contrast, we found that this pattern has been disrupted in AD brains (Fig. 2B). For instance, the inhibitory and excitatory neurons demonstrated mixed latent communication patterns (e.g., Chandelier cells have been grouped into excitatory patterns). In addition, the major driving signal pathways for different cell types also changed noticeably. For example, the WNT pathways became one major contributor to the excitatory group, while ANGPT switched from major contributors in non-neuronal cells to the inhibitory group. Together, these results suggested extensive alterations in global C2C communication patterns and signaling usage in the outgoing network. The incoming network exhibits similar disruptions albeit with slight differences, with more pathways being grouped with the excitatory pattern (Fig. S5). In summary, global communication pattern analysis reveals the irregular inter-mixing of cell types and signaling pathways in AD brains.

### Pairwise cell type C2C comparison highlights disturbed communication strength across various cell types in AD brains

**Fig. 3:**
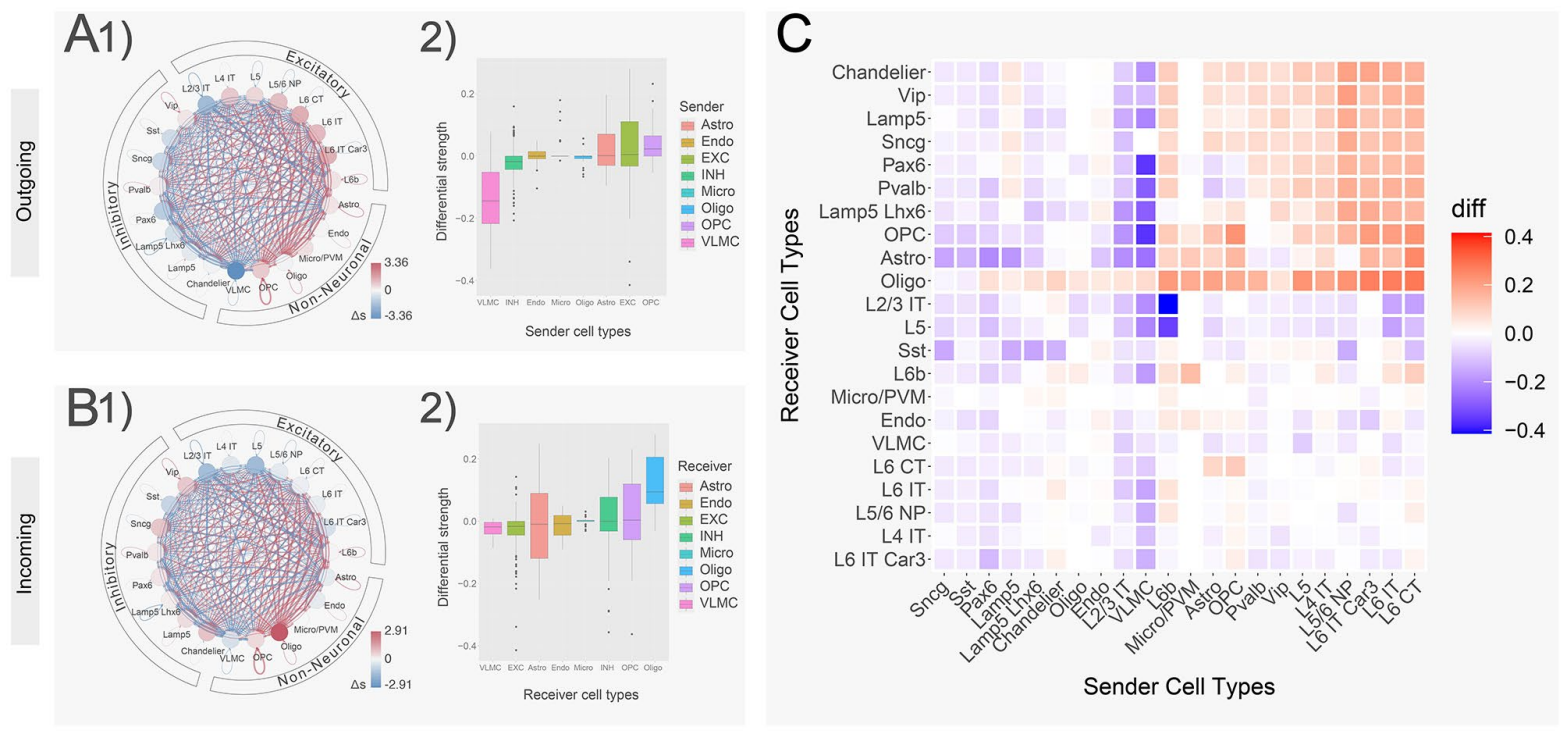
Cell-type centric cell-to-cell communication analysis. (**A**) Differential network and boxplot of the outgoing cell-to-cell communication between Alzheimer’s and control. The nodes of the network were colored by the difference aggregated across signaling pathways. Red indicates an increased communication in Alzheimer’s (while blue indicates decreased). (**B**) Similar to A. Differential network and boxplot of the incoming cell-to-cell communication between Alzheimer’s and control. (**C**) Clustered heatmap of the cell-to-cell communication between all pair-wise cell types. Red indicates an increased communication in Alzheimer’s (while blue indicates decreased)

After checking the global C2C pattern perturbations, we focused on cell-type-centric communication changes by aggregating all Ligand receptor pairs in our 3D C2C matrix. In our C2C network, each edge (aka communication strength) is a product of the sender’s ligand gene expression with the receiver’s receptor gene expression. First, we calculated the overall outgoing/incoming communication strength ($$ {s}_{i}$$) for a particular cell type $$ i$$ by aggregating all outgoing/incoming edges for that cell type. We then calculated the difference ($$ \varDelta {s}_{i}$$) between AD and control samples. Interestingly, we found that AD brains showed noticeably increased outgoing communication strength in most excitatory cell types ranging from 0.37 to 1.34 std dev above the collective mean, except for L2/3 IT. On the other hand, the inhibitory group showed a decrease of communication strength ranging from − 0.13 to -1.17 std dev below the mean (Fig. 3A1, Table S1). In the non-neuronal cells, Astro, OPC, and Micro/PVM also showed a higher level of outgoing communication strength (0.35, 0.79, 0.44 std dev above, Fig. 3A1). Since each node in the circle plot represents a summation across all ligand-receptor pathways, we also made a boxplot of each individual ligand-receptor pathway for each cell type. Agreeing with the intensely bluely-colored “summed” node in its circle plot, the VLMC showed that more than 75% of its ligand-receptor pathways are down in Alzheimer’s compared to those of control (Fig. 3A2, Table S2).

On the other hand, in the incoming network, we found that the incoming communication strength is decreased in all excitatory cell types, showing a reduction ranging from − 0.05 to -1.76 std dev below the collective mean. In comparison, there is a general increase in the inhibitory group showing an increase ranging from 0.20 to 1.02 std dev above the mean, except for SST (Fig. 3B1, Table S3). In the non-neuronal cells, Astro, Endo, and VLMC also showed a lower level of incoming communications (-0.36, -0.35, -0.64 std dev below), except for a strong increase in Oligo (3.09 std dev above, Fig. 3B1). Agreeing with the intensely redly-colored “summed” node in its circle plot, the Oligo showed that more than 90% of its ligand-receptor pathways are up in Alzheimer’s compared to those of control (Fig. 3B2, Table S4). The different results of these incoming and outgoing network comparisons among cell classes show that intercellular signaling is regulated in a cell-type-specific manner in AD.

Next, we calculated the pair-wise AD-to-normal C2C communication, aiming to find the major driver cell types to explain the above changes. As shown in Fig. 3C, we found various cell types demonstrated distinct patterns of C2C disruption. Specifically, excitatory neurons demonstrated more targeted disruption in C2C communication patterns, while inhibitory neurons and non-neuronal cells demonstrated more global disruptions. For example, the excitatory neuron groups significantly increased their communication mostly to inhibitory neurons (median $\varDelta {s}_{i}$ 0.101, upper right red quadrant in Fig. 3C), but kept a similar level of communication to other cell types (median $\varDelta {s}_{i}$ -0.006, bottom right quadrant in Fig. 3C). In contrast, most inhibitory neurons and non-neuronal cells globally decreased their communication strengths to almost all cell types (median $\varDelta {s}_{i}$ -0.034, left side of the heatmap in Fig. 3C). Our findings add to previous reports of AD patients showing an increased excitatory to inhibitory synaptic ratio by considering now their cross-cell type communication [53, 54].

### Canonical neuroinflammation and neuroprotection signaling pathways in AD are dis-regulated in a cell-type-specific manner

**Fig. 4:**
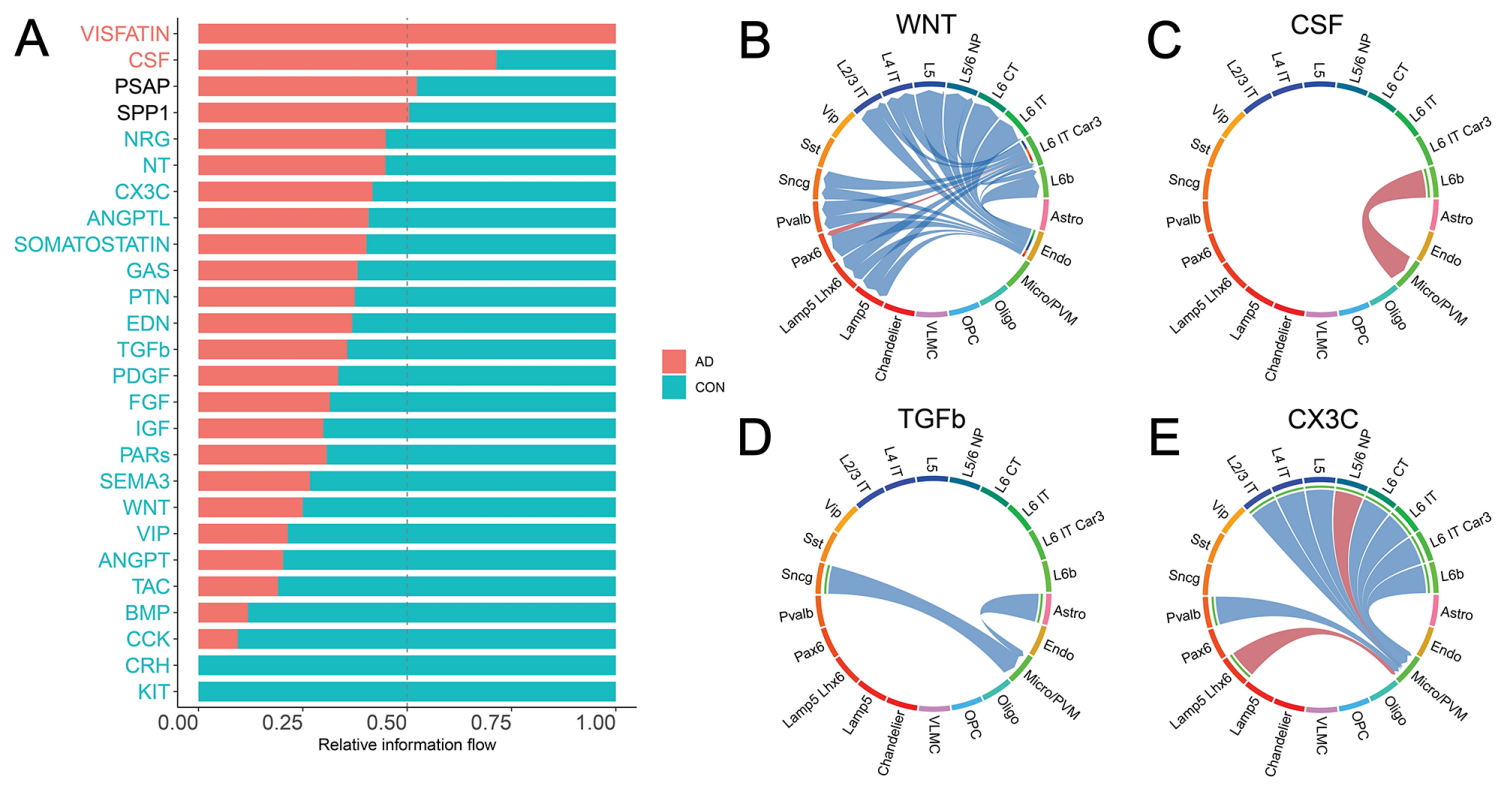
Signaling pathways of cell-to-cell communication analysis. (**A**) Comparison of the signaling pathway flow between Alzheimer’s and control. The flow is defined as the summation of the ligand-receptor gene expression products of that specific pathway across all sender-receiver pairs. Red indicates an increased communication in Alzheimer’s (while blue indicates decreased). (**B**) Communication strength difference among cell types in the pathway WNT. Red indicates an increased communication in Alzheimer’s (while blue indicates decreased). (**C**) Communication strength difference among cell types in the pathway CSF. (**D**) Communication strength difference among cell types in the pathway TGFβ. (**E**) Communication strength difference among cell types in the pathway CX3C

Our previous analyses mainly focused on the cell-type-level communication strength perturbations in the C2C network comparison without considering the impact of their communication pathways. To fill this gap, we also performed a signaling-pathway-centric analysis by evaluating the contribution of all involved ligand-receptor pairs (details in Methods 2.3 C). Each signaling pathway contains multiple ligand-receptor gene pairs. As shown in Fig. 4A, many robustly expressed pathways in the human brain demonstrated significantly altered involvement in C2C communication network. For example, for each pathway, we aggregated the communication strength of all involved Ligand Receptor pairs and across all cell type pairs. After a Wilcoxon Rank Sum test, we found that 22 pathways showed significantly decreased communication activity, while 2 pathways demonstrated increased activity (Fig. 4A). We chose to focus on 4 canonical ligand-receptor interactions with strong literature support for further analysis, namely the WNT, CSF, TGFβ, and CX3C pathways (Fig. 4B-E) [22, 23, 55, 56].

Neuronal inflammation plays a significant role in the AD pathology [57, 58]. Consistently, we found that two inflammation-related pathways WNT and CSF are dysregulated in AD. For example, the WNT signaling pathway plays multifaceted roles in CNS diseases by modulating neuroimmune interactions [55]. We found that the WNT pathway has significantly reduced its involvement in C2C communication (30% of control, *P* = 2.086e-7, Fig. 4A, Table S5), driven by decrease of communication from endothelial senders to neuronal receivers (Fig. 4B). Mechanistically, the downregulation of the WNT ligand gene can cause overactivity of the lithium-targeted GSK3β enzyme, leading to changes in neurogenesis, inflammation, oxidative stress, and circadian dysregulation in neuronal cell types [25]. Additionally, lines of literature also report the CSF pathway as a well-known inflammatory pathway primarily involving microglia [46, 59–61]. Consistently, we found that the CSF pathway has been significantly upregulated in AD patients (250% of control, *P* = 0, Fig. 4A). Such increased involvement is mainly driven by the increased communication from the excitatory neurons L6b to Microglia cells (Fig. 4C). The disturbance of these inflammatory pathways provides a cross cell-type mechanistic insight to AD pathology.

Next, we move on to neuroprotective signaling pathways, specifically TGFβ and CX3C. We observed the downregulation of TGFβ signaling in Alzheimer’s in the communication to Micro/PVM cell type (60% of control, *P* = 0, Fig. 4A and D, Table S5). A decrease in TGFβ1 has been associated with a higher burden of Aβ in the parenchyma, which correlates with an increased microglia activation [62]. The suppression of the neuroprotective role of the signaling pathway TGFβ1 against Aβ toxicity in the diseased cell types may mechanistically explain Alzheimer’s disease. Adding on, we also found the decrease of another neuroprotective signaling pathway, CX3C (70% of control, *P* = 4.883e-2, Fig. 4A). CX3CL1 has been demonstrated to play a neuroprotective role in CNS by reducing neurotoxicity from microglial activation [63]. Our C2C analysis provides a more detailed picture than that existing in the literature by seeing that communication is directed to the Micro/PVM cell type from excitatory neurons (Fig. 4E). In summary, we discover that both the signaling pathways that cause neuroinflammation and those that protect against it are regulated in a cell-type-specific manner. The respective increase and decrease of these pathways may mechanistically explain Alzheimer’s Disease in a cross-cell type manner.

### Intracellular cell-to-cell communication analysis reveals a strong connection to neuroinflammatory AD risk genes

**Fig. 5:**
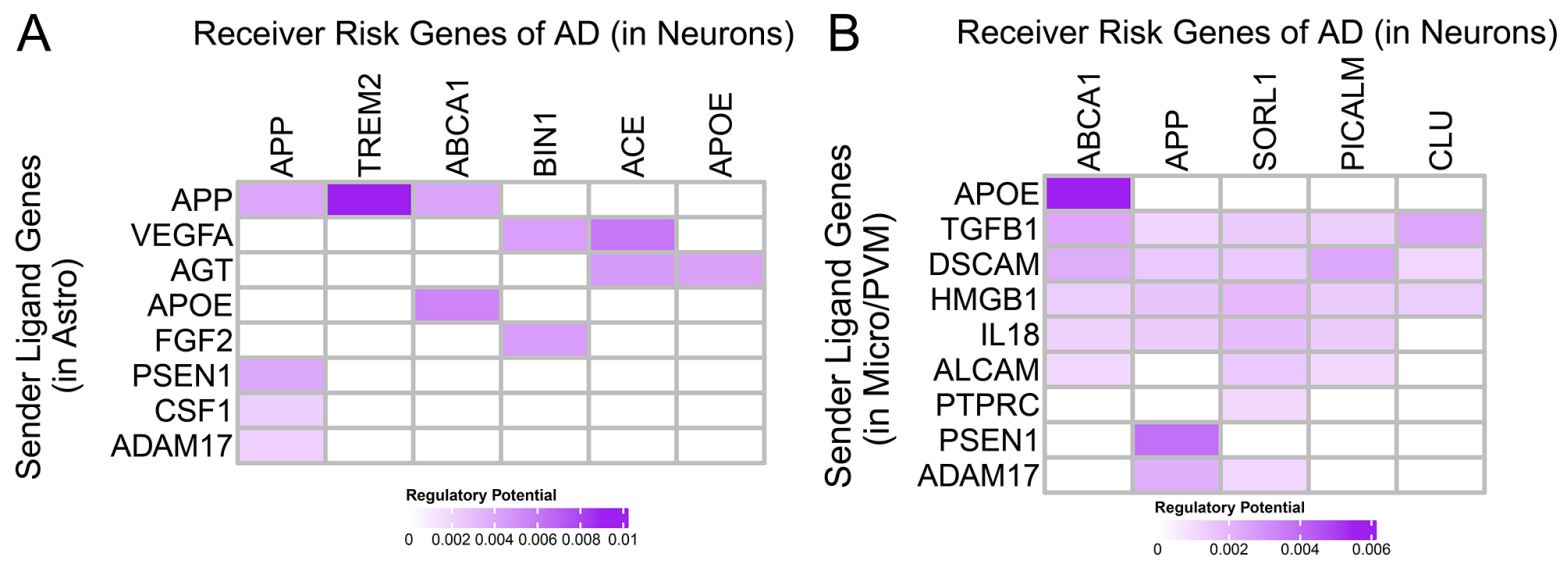
Connection to intracellular risk genes. (**A**) Connection of astrocyte’s ligand genes to neurons’ risk genes in Alzheimer’s. (**B**) Connection of microglia/perivascular macrophages’ ligand genes to neurons’ risk genes in Alzheimer’s. More intensely colored boxes indicate a stronger regulation

Finally, we seek to see how extracellular signaling is connected with well-known AD risk genes in an intra-cellular manner (Table S6). To accomplish this, we first extracted AD risk genes, including APP, ABCA1, and TREM2 (details see Methods 2.4, Fig. S3). Then, we defined Ligand-to-risk-gene regulation scores by combining C2C communication networks outside cells and gene-gene interaction networks within cells via NicheNet [42]. Since many of the well-known disease risk genes appear to be regulated in a cell type-specific fashion by our extracellular cell-to-cell communication analysis above, we considered only a subset of sending cell types and receiving cell types (Fig. 5). Specifically, we set astrocytes and microglia as the senders and neurons as the receivers to find signals that cause neuron dysfunctions or neuronal death.

In the astrocyte-to-neuron signaling, we find ligand-target links connecting neurological risk genes to potential upstream effectors, such as the APP-TREM2 (Regulatory Score 0.00904, z-score 21.44 compared to all documented ligand-target links, the maximum in heatmap shown) and APP-ABCA1 link (Regulatory Score 0.00410, z-score 9.10) (Fig. 5A). TREM2 expression in microglia and macrophages results in decreased phagocytosis of apoptotic neurons, increasing Aβ accumulation in AD Phenotype [46, 64, 65]. Additionally, ABCA1 deficiency increases amyloid deposition in the brains of amyloid precursor protein (APP) transgenic mice [66, 67]. In the microglia-to-neuron signaling, we find ligand-target links such as APOE-ABCA1 (Regulatory Score 0.005490, z-score 12.57, the maximum in heatmap shown), and PSEN1-APP (Regulatory Score 0.003858, z-score 8.49) (Fig. 5B). PSEN1 and PSEN2 mutations have been linked with the Amyloid protein precursor in early-onset Alzheimer disease [68]. By linking our extracellular signaling above to intracellular risk genes, our ligand-target analysis completes our cell-to-cell communication analysis and supports our hypothesis that AD communication dysregulations happen with cell-type-specificity and that improper regulation of extracellular signaling genes is linked to intracellular risk genes.

## Discussion

Alzheimer’s Disease is a neurological disorder involving genetic, epigenetic, and environmental factors through various processes. The improper regulation of cell-cell signaling can be linked to Alzheimer’s disease [69–71]. With the explosion of data in the recent consortium initiatives and the new technological developments in single-cell sequencing, we are able to, for the first time, systematically compare cell-cell communications in Alzheimer’s and control brains.

Results from our C2C analysis have shown that there is a global C2C communication pattern intermixing (inhibitory Chandelier cells in the excitatory group Fig. 2) and signaling pathway misusage in AD brains (e.g. ANGPT, WNT pathways, Fig. 2). Additionally, we also observed a large degree of C2C communication disruption heterogeneity across various cell types. For example, excitatory neurons tend to solely increase their communication strength with inhibitory neurons, while non-neuronal cells and inhibitory neurons globally decrease their communication to most cell types (Fig. 3). This signifies the importance of employing single-cell technologies in AD studies to dissect the extensive genetic heterogeneity in complex tissues like the human brain. Furthermore, we highlighted the involvement of the neural inflammatory and neural protective pathways, such as WNT, CSF, TGFβ, and CX3C, in AD patients (Fig. 4). Their disturbed behavior can pass erroneous information both inter- and intra-cellularly to directly impact well-known AD risk genes (Fig. 5).

In this study, cell-cell communication is inferred from the expression of protein-coding genes, thus not fully capturing other signaling events in the brain, such as nonprotein molecules like neurotransmitters. We seek to address this limitation in the future by considering also the gene expression of the neurotransmitter-synthesis and transporter proteins to study more thoroughly the communication networks of the brain. In our last risk gene analysis, we can also provide a finer resolution of our single cells by incorporating chromatin-accessibility analysis scATAC-seq [72]. Finally, future work could be directed to include Braak staging to understand the time-specific changes in cell-cell communication in AD.

Despite these limitations, we believe our work can serve as a valuable first step in investigating the intercellular molecular mechanisms underlying Alzheimer’s disease beyond the general bulk-sequencing and isolated cellular picture. We have made our computational pipeline publicly available for all researchers (https://github.com/dssikdar/C2Cv0.git). With further technological advances and community efforts for population-scale single-cell sequencing, we expect exponentially increased power to accurately quantify C2C communication and its alterations in AD brains, hoping for the subsequent alleviation of the pain caused by Alzheimer’s Disease.

## Conclusions

In our study, we conducted an extensive bioinformatics analysis to explore changes in cell-cell communication within the brains of individuals with Alzheimer’s Disease (AD). We identified several intercellular signaling pathways and cell types that appear to be modified in AD. Notably, genes within these altered pathways demonstrate a substantial link to intracellular molecular pathways, which we believe are likely disrupted across various brain cell types in AD. This suggests that more studies examining cross-cell type effects can be valuable for understanding Alzheimer’s pathogenesis.

### Electronic supplementary material

Below is the link to the electronic supplementary material.

Supplementary Material 1

## Data Availability

No new experimental data were acquired in this study, and the snRNA-seq data are publicly available at (https://adknowledgeportal.synapse.org/) with a Synapse license (SynID: syn18485175).

## Abbreviations

AD: Alzheimer’s Disease
Astro: Astrocytes
BICCN: BRAIN Initiative Cell Census Network
C2C: Cell-to-cell Communication
CON: Control
CT: Cortico-thalamic
Endo: Endothelial cells
GWAS: Genome-Wide Association Studies
IT: Intra-telencephalic
L#: Signifies the cortical layer context
Micro/PVM: Microglia/Perivascular macrophages
NP: Near-projecting
Oligo: Oligodendrocytes
OPC: Oligodendrocyte precursor cells
PFC: Human prefrontal cortex
snRNA-seq: Single-nucleus RNA sequencing
VLMC: Vascular Leptomeningeal cells
WES: Whole Exome Sequencing

## References

[1] Masters CL, Bateman R, Blennow K, Rowe CC, Sperling RA, Cummings JL (2015). Alzheimer’s disease. Nat Rev Dis Primers.

[2] Hardy J, Selkoe DJ (2002). The amyloid hypothesis of Alzheimer’s disease: progress and problems on the road to therapeutics. Science.

[3] Braak H, Braak E (1991). Neuropathological stageing of Alzheimer-related changes. Acta Neuropathol.

[4] Castro DM, Dillon C, Machnicki G, Allegri RF (2010). The economic cost of Alzheimer’s disease: family or public health burden?. Dement Neuropsychol.

[5] Sloane PD, Zimmerman S, Suchindran C, Reed P, Wang L, Boustani M, Sudha S (2002). The public health impact of Alzheimer’s disease, 2000–2050: potential implication of treatment advances. Annu Rev Public Health.

[6] Fisher L, Lieberman MA (1994). Alzheimer’s disease: the impact of the family on spouses, offspring, and inlaws. Fam Process.

[7] De Strooper B, Karran E (2016). The Cellular phase of Alzheimer’s Disease. Cell.

[8] Lambert E, Saha O, Soares Landeira B, Melo de Farias AR, Hermant X, Carrier A, Pelletier A, Gadaut J, Davoine L, Dupont C (2022). The Alzheimer susceptibility gene BIN1 induces isoform-dependent neurotoxicity through early endosome defects. Acta Neuropathol Commun.

[9] Zhong S, Zhang S, Fan X, Wu Q, Yan L, Dong J, Zhang H, Li L, Sun L, Pan N (2018). A single-cell RNA-seq survey of the developmental landscape of the human prefrontal cortex. Nature.

[10] Lake BB, Chen S, Sos BC, Fan J, Kaeser GE, Yung YC, Duong TE, Gao D, Chun J, Kharchenko PV (2018). Integrative single-cell analysis of transcriptional and epigenetic states in the human adult brain. Nat Biotechnol.

[11] Habib N, Avraham-Davidi I, Basu A, Burks T, Shekhar K, Hofree M, Choudhury SR, Aguet F, Gelfand E, Ardlie K (2017). Massively parallel single-nucleus RNA-seq with DroNc-seq. Nat Methods.

[12] Lake BB, Ai R, Kaeser GE, Salathia NS, Yung YC, Liu R, Wildberg A, Gao D, Fung HL, Chen S (2016). Neuronal subtypes and diversity revealed by single-nucleus RNA sequencing of the human brain. Science.

[13] Macosko EZ, Basu A, Satija R, Nemesh J, Shekhar K, Goldman M, Tirosh I, Bialas AR, Kamitaki N, Martersteck EM (2015). Highly parallel genome-wide expression profiling of individual cells using Nanoliter droplets. Cell.

[14] Mathys H, Davila-Velderrain J, Peng Z, Gao F, Mohammadi S, Young JZ, Menon M, He L, Abdurrob F, Jiang X (2019). Single-cell transcriptomic analysis of Alzheimer’s disease. Nature.

[15] Morabito S, Miyoshi E, Michael N, Shahin S, Martini AC, Head E, Silva J, Leavy K, Perez-Rosendahl M, Swarup V (2021). Single-nucleus chromatin accessibility and transcriptomic characterization of Alzheimer’s disease. Nat Genet.

[16] Spill F, Reynolds DS, Kamm RD, Zaman MH (2016). Impact of the physical microenvironment on tumor progression and metastasis. Curr Opin Biotechnol.

[17] Bloom AB, Zaman MH (2014). Influence of the microenvironment on cell fate determination and migration. Physiol Genomics.

[18] Alberts B, Hunt T, Johnson A, Lewis J, Morgan D, Raff MC, Roberts K, Walter P, Wilson JH, Roberts K (2015). Molecular biology of the cell, Sixth edition.

[19] Mellman I, Nelson WJ (2008). Coordinated protein sorting, targeting and distribution in polarized cells. Nat Rev Mol Cell Biol.

[20] Nelson CM, Bissell MJ (2006). Of extracellular matrix, scaffolds, and signaling: tissue architecture regulates development, homeostasis, and cancer. Annu Rev Cell Dev Biol.

[21] Ecker JR, Geschwind DH, Kriegstein AR, Ngai J, Osten P, Polioudakis D, Regev A, Sestan N, Wickersham IR, Zeng H (2017). The BRAIN Initiative Cell Census Consortium: lessons learned toward Generating a Comprehensive Brain Cell Atlas. Neuron.

[22] Pons V, Lévesque P, Plante MM, Rivest S (2021). Conditional genetic deletion of CSF1 receptor in microglia ameliorates the physiopathology of Alzheimer’s disease. Alzheimers Res Ther.

[23] Chen P, Zhao W, Guo Y, Xu J, Yin M. CX3CL1/CX3CR1 in Alzheimer’s disease: a target for neuroprotection. Biomed Res Int. 2016;2016:8090918. 10.1155/2016/8090918 PMC4939332 27429982

[24] Caraci F, Battaglia G, Bruno V, Bosco P, Carbonaro V, Giuffrida ML, Drago F, Sortino MA, Nicoletti F, Copani A (2011). TGF-β1 pathway as a new target for neuroprotection in Alzheimer’s disease. CNS Neurosci Ther.

[25] Palomer E, Buechler J, Salinas PC (2019). Wnt signaling deregulation in the aging and Alzheimer’s brain. Front Cell Neurosci.

[26] Zheng GXY, Terry JM, Belgrader P, Ryvkin P, Bent ZW, Wilson R, Ziraldo SB, Wheeler TD, McDermott GP, Zhu J (2017). Massively parallel digital transcriptional profiling of single cells. Nat Commun.

[27] Fleming SJ, Chaffin MD, Arduini A, Akkad AD, Banks E, Marioni JC, Philippakis AA, Ellinor PT, Babadi M (2023). Unsupervised removal of systematic background noise from droplet-based single-cell experiments using CellBender. Nat Methods.

[28] Rath S, Sharma R, Gupta R, Ast T, Chan C, Durham TJ, Goodman RP, Grabarek Z, Haas ME, Hung WHW (2020). MitoCarta3.0: an updated mitochondrial proteome now with sub-organelle localization and pathway annotations. Nucleic Acids Res.

[29] Hodge RD, Bakken TE, Miller JA, Smith KA, Barkan ER, Graybuck LT, Close JL, Long B, Johansen N, Penn O (2019). Conserved cell types with divergent features in human versus mouse cortex. Nature.

[30] Wolock SL, Lopez R, Klein AM (2019). Scrublet: computational identification of cell doublets in single-cell Transcriptomic Data. Cell Syst.

[31] Gayoso A, Shor J. JonathanShor/DoubletDetection: doubletdetection v4.2. 2022.

[32] Li B, Gould J, Yang Y, Sarkizova S, Tabaka M, Ashenberg O, Rosen Y, Slyper M, Kowalczyk MS, Villani AC (2020). Cumulus provides cloud-based data analysis for large-scale single-cell and single-nucleus RNA-seq. Nat Methods.

[33] Korsunsky I, Millard N, Fan J, Slowikowski K, Zhang F, Wei K, Baglaenko Y, Brenner M, Loh P-R, Raychaudhuri S (2019). Fast, sensitive and accurate integration of single-cell data with Harmony. Nat Methods.

[34] Ma S, Skarica M, Li Q, Xu C, Risgaard RD, Tebbenkamp ATN, Mato-Blanco X, Kovner R, Krsnik Ž, de Martin X (2022). Molecular and cellular evolution of the primate dorsolateral prefrontal cortex. Science.

[35] Jin S, Guerrero-Juarez CF, Zhang L, Chang I, Ramos R, Kuan C-H, Myung P, Plikus MV, Nie Q (2021). Inference and analysis of cell-cell communication using CellChat. Nat Commun.

[36] Huuki-Myers L, Spangler A, Eagles N, Montgomery KD, Kwon SH, Guo B, Grant-Peters M, Divecha HR, Tippani M, Sriworarat C et al. Integrated single cell and unsupervised spatial transcriptomic analysis defines molecular anatomy of the human dorsolateral prefrontal cortex. bioRxiv. 2023.

[37] Brunet J-P, Tamayo P, Golub TR, Mesirov JP (2004). Metagenes and molecular pattern discovery using matrix factorization. Proc Natl Acad Sci USA.

[38] Bellenguez C, Küçükali F, Jansen IE, Kleineidam L, Moreno-Grau S, Amin N, Naj AC, Campos-Martin R, Grenier-Boley B, Andrade V (2022). New insights into the genetic etiology of Alzheimer’s disease and related dementias. Nat Genet.

[39] Wightman DP, Jansen IE, Savage JE, Shadrin AA, Bahrami S, Holland D, Rongve A, Børte S, Winsvold BS, Drange OK (2021). A genome-wide association study with 1,126,563 individuals identifies new risk loci for Alzheimer’s disease. Nat Genet.

[40] Holstege H, Hulsman M, Charbonnier C, Grenier-Boley B, Quenez O, Grozeva D, van Rooij JGJ, Sims R, Ahmad S, Amin N (2022). Exome sequencing identifies rare damaging variants in ATP8B4 and ABCA1 as risk factors for Alzheimer’s disease. Nat Genet.

[41] Hu Y-S, Xin J, Hu Y, Zhang L, Wang J (2017). Analyzing the genes related to Alzheimer’s disease via a network and pathway-based approach. Alzheimers Res Ther.

[42] Browaeys R, Saelens W, Saeys Y (2020). NicheNet: modeling intercellular communication by linking ligands to target genes. Nat Methods.

[43] Yim A, Smith C, Brown AM (2022). Osteopontin/secreted phosphoprotein-1 harnesses glial-, immune-, and neuronal cell ligand-receptor interactions to sense and regulate acute and chronic neuroinflammation. Immunol Rev.

[44] Hampton DW, Asher RA, Kondo T, Steeves JD, Ramer MS, Fawcett JW (2007). A potential role for bone morphogenetic protein signalling in glial cell fate determination following adult central nervous system injury in vivo. Eur J Neurosci.

[45] Kumar A, Rassoli A, Raizada MK (1988). Angiotensinogen gene expression in neuronal and glial cells in primary cultures of rat brain. J Neurosci Res.

[46] He L, Shi H, Zhang G, Peng Y, Ghosh A, Zhang M, Hu X, Liu C, Shao Y, Wang S (2023). A novel CCK receptor GPR173 mediates potentiation of GABAergic Inhibition. J Neurosci.

[47] Miklós IH, Kovács KJ (2002). GABAergic innervation of corticotropin-releasing hormone (CRH)-secreting parvocellular neurons and its plasticity as demonstrated by quantitative immunoelectron microscopy. Neuroscience.

[48] Tallent MK, Siggins GR (1997). Somatostatin depresses excitatory but not inhibitory neurotransmission in rat CA1 hippocampus. J Neurophysiol.

[49] Goyal RK, Rattan S, Said SI (1980). VIP as a possible neurotransmitter of non-cholinergic non-adrenergic inhibitory neurones. Nature.

[50] Pasterkamp RJ, Giger RJ (2009). Semaphorin function in neural plasticity and disease. Curr Opin Neurobiol.

[51] Nandi S, Gokhan S, Dai XM, Wei S, Enikolopov G, Lin H, Mehler MF, Stanley ER (2012). The CSF-1 receptor ligands IL-34 and CSF-1 exhibit distinct developmental brain expression patterns and regulate neural progenitor cell maintenance and maturation. Dev Biol.

[52] Blum R, Konnerth A (2005). Neurotrophin-mediated rapid signaling in the central nervous system: mechanisms and functions. Physiol (Bethesda).

[53] Ranasinghe KG, Verma P, Cai C, Xie X, Kudo K, Gao X, Lerner H, Mizuiri D, Strom A, Iaccarino L et al. Altered excitatory and inhibitory neuronal subpopulation parameters are distinctly associated with tau and amyloid in Alzheimer’s disease. Elife. 2022;11. 10.7554/eLife.77850 PMC9217132 35616532

[54] Lauterborn JC, Scaduto P, Cox CD, Schulmann A, Lynch G, Gall CM, Keene CD, Limon A (2021). Increased excitatory to inhibitory synaptic ratio in parietal cortex samples from individuals with Alzheimer’s disease. Nat Commun.

[55] Marchetti B, Pluchino S (2013). Wnt your brain be inflamed? Yes, it wnt!. Trends Mol Med.

[56] von Bernhardi R, Cornejo F, Parada GE, Eugenín J (2015). Role of TGFβ signaling in the pathogenesis of Alzheimer’s disease. Front Cell Neurosci.

[57] Frautschy SA, Baird A, Cole GM (1991). Effects of injected Alzheimer beta-amyloid cores in rat brain. Proc Natl Acad Sci U S A.

[58] Cai Z, Hussain MD, Yan LJ (2014). Microglia, neuroinflammation, and beta-amyloid protein in Alzheimer’s disease. Int J Neurosci.

[59] Pons V, Lévesque P, Plante M-M, Rivest S (2021). Conditional genetic deletion of CSF1 receptor in microglia ameliorates the physiopathology of Alzheimer’s disease. Alzheimers Res Ther.

[60] Esaulova E, Cantoni C, Shchukina I, Zaitsev K, Bucelli RC, Wu GF, Artyomov MN, Cross AH, Edelson BT. Single-cell RNA-seq analysis of human CSF microglia and myeloid cells in neuroinflammation. Neurol Neuroimmunol Neuroinflamm. 2020;7(4). 10.1212/NXI.0000000000000732 PMC7217663 32371549

[61] Imai Y, Kohsaka S (2002). Intracellular signaling in M-CSF-induced microglia activation: role of Iba1. Glia.

[62] von Bernhardi R, Cornejo F, Parada GE, Eugenín J (2015). Role of TGFβ signaling in the pathogenesis of Alzheimer’s disease. Front Cell Neurosci.

[63] Chen P, Zhao W, Guo Y, Xu J, Yin M. CX3CL1/CX3CR1 in Alzheimer’s disease: a target for neuroprotection. BioMed research international. 2016;2016:8090918–8090918. 10.1155/2016/8090918 PMC4939332 27429982

[64] Liu W, Taso O, Wang R, Bayram S, Graham AC, Garcia-Reitboeck P, Mallach A, Andrews WD, Piers TM, Botia JA (2020). Trem2 promotes anti-inflammatory responses in microglia and is suppressed under pro-inflammatory conditions. Hum Mol Genet.

[65] Hickman SE, El Khoury J (2014). TREM2 and the neuroimmunology of Alzheimer’s disease. Biochem Pharmacol.

[66] Fitz NF, Cronican AA, Saleem M, Fauq AH, Chapman R, Lefterov I, Koldamova R (2012). Abca1 deficiency affects Alzheimer’s disease-like phenotype in human ApoE4 but not in ApoE3-targeted replacement mice. J Neurosci.

[67] Wahrle SE, Jiang H, Parsadanian M, Kim J, Li A, Knoten A, Jain S, Hirsch-Reinshagen V, Wellington CL, Bales KR (2008). Overexpression of ABCA1 reduces amyloid deposition in the PDAPP mouse model of Alzheimer disease. J Clin Invest.

[68] Lanoiselée HM, Nicolas G, Wallon D, Rovelet-Lecrux A, Lacour M, Rousseau S, Richard AC, Pasquier F, Rollin-Sillaire A, Martinaud O et al. APP, PSEN1, and PSEN2 mutations in early-onset Alzheimer disease: a genetic screening study of familial and sporadic cases. PLoS Med. 2017;14(3):e1002270. 10.1371/journal.pmed.1002270 PMC5370101 28350801

[69] Calvo-Rodriguez M, Bacskai BJ (2021). Mitochondria and Calcium in Alzheimer’s Disease: from cell signaling to neuronal cell death. Trends Neurosci.

[70] Ho GJ, Drego R, Hakimian E, Masliah E (2005). Mechanisms of cell signaling and inflammation in Alzheimer’s disease. Curr Drug Targets Inflamm Allergy.

[71] Mattson MP, Barger SW, Furukawa K, Bruce AJ, Wyss-Coray T, Mark RJ, Mucke L (1997). Cellular signaling roles of TGF beta, TNF alpha and beta APP in brain injury responses and Alzheimer’s disease. Brain Res Brain Res Rev.

[72] Buenrostro JD, Wu B, Litzenburger UM, Ruff D, Gonzales ML, Snyder MP, Chang HY, Greenleaf WJ (2015). Single-cell chromatin accessibility reveals principles of regulatory variation. Nature.

