# Supplementary material for: Characterizing dysregulations via cell-cell communications in Alzheimer’s brains using single-cell transcriptomes
Source: BMC Neurosci. 2024 May 13;25:24. doi: 10.1186/s12868-024-00867-y (PMC11089696; doi:10.1186/s12868-024-00867-y)
Supplement: Supplementary file 1 — Supplementary Material 1 [file 12868_2024_867_MOESM1_ESM.docx]

Supplementary Notes for Characterizing dysregulations via cell-cell communications in Alzheimer's brains using single-cell transcriptomes

Che Yu Lee^1 ¶^, Dylan Riffle^1 ¶^, Yifeng Xiong^1 ¶^, Nadia Momtaz^2^, Yutong Lei^1^, Joseph M. Pariser^1^, Diptanshu Sikdar^1^, Ahyeon Hwang^1, 3^, Ziheng Duan^1^, Jing Zhang^1^ *

^1^ Department of Computer Science, University of California, Irvine, CA, USA

^2^ Department of Biomedical Engineering, Johns Hopkins University, Baltimore, MD, USA

^3^ Mathematical, Computational and Systems Biology, University of California, Irvine, CA, USA

*To whom correspondence should be addressed.

Table of Contents

[Title Page 1](#_Toc159444603)

[Supplementary Figures 3](#_Toc159444604)

[Figure S1. Marker Genes for the Sub Cell Types. 3](#_Toc159444605)

[Figure S2. Images of the Spatial Data. 4](#_Toc159444606)

[Figure S3. Venn Diagram of Risk Gene Extraction. 5](#_Toc159444607)

[Figure S4. Barplots of cell numbers and ligand-receptor interaction counts. 6](#_Toc159444608)

[Figure S5. Pattern Analysis of the Incoming Cell-to-Cell Communication. 7](#_Toc159444609)

[Figure S6. Organization of our GitHub Repository. 8](#_Toc159444610)

[Supplementary Tables 9](#_Toc159444611)

[Table S1. Aggregated Communication for the Outgoing Network. 9](#_Toc159444612)

[Table S2. Distribution of Communication for the Outgoing Network. 10](#_Toc159444613)

[Table S3. Aggregated Communication for the Incoming Network. 11](#_Toc159444614)

[Table S4. Distribution of Communication for the Incoming Network. 12](#_Toc159444615)

[Table S5. Ratio and P-values of each Communication Pathway. 13](#_Toc159444616)

[Table S6. Alzheimer’s Risk Genes. 14](#_Toc159444617)

[Table S7. Cell and Ligand-Receptor Count Correlation. 15](#_Toc159444618)

[Supplementary Methods 16](#_Toc159444619)

[Methods S1. QC and Preprocessing. 16](#_Toc159444620)

# Supplementary Figures

## **Figure S1.** Marker Genes for the Sub Cell Types.


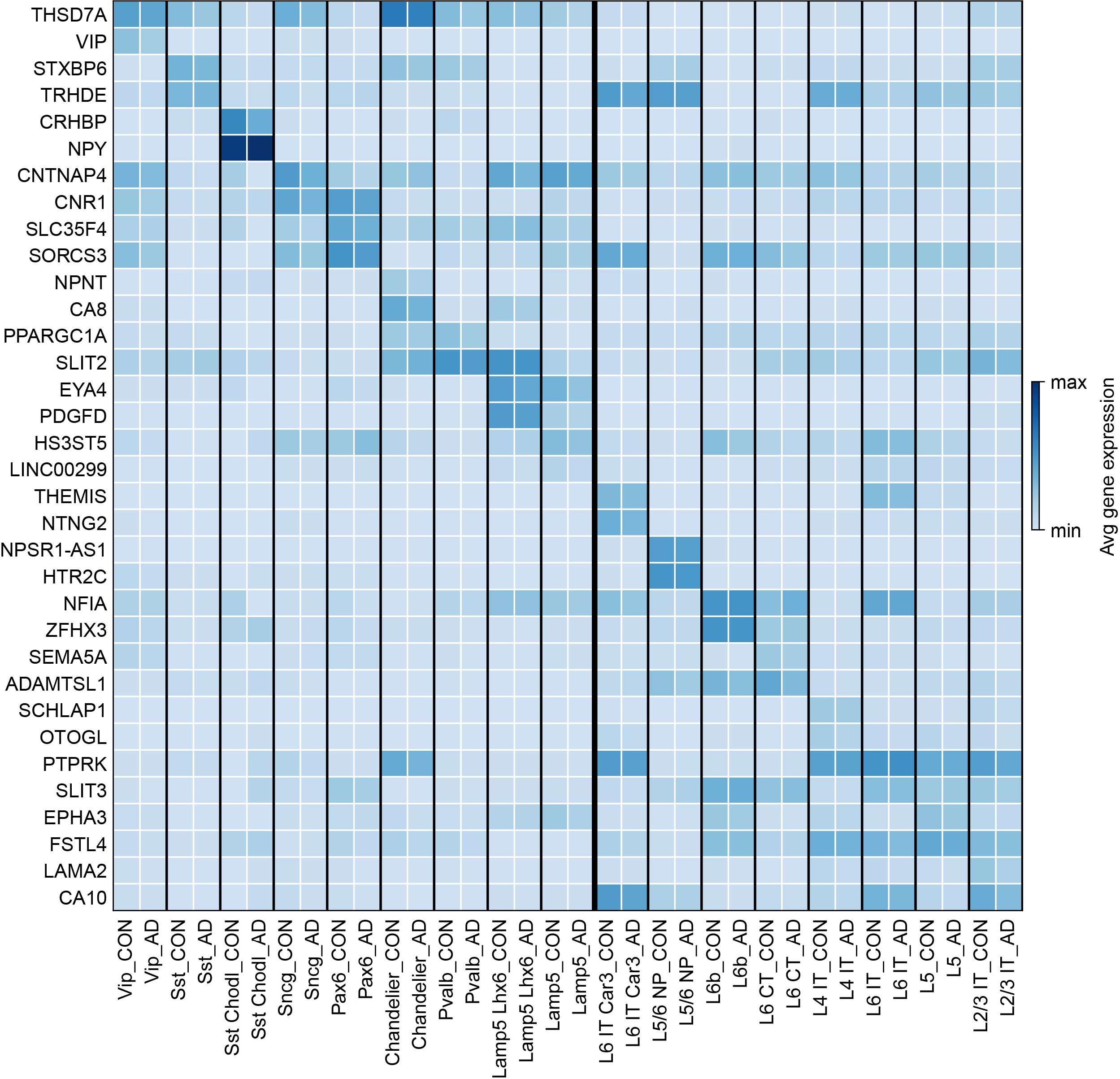


We utilized the above marker genes to further label the neuronal sub-cell-types.

## **Figure S2.** Images of the Spatial Data.


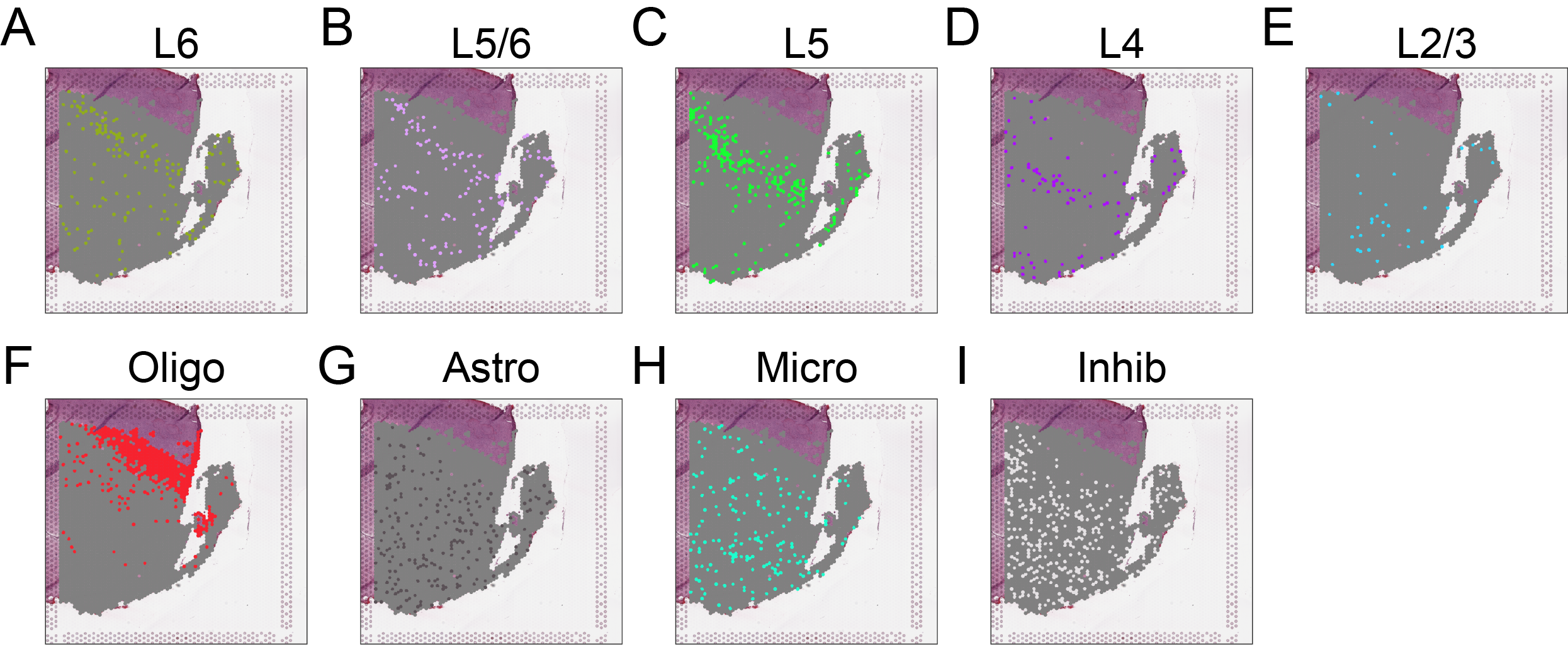


We show the layer-specificity of certain cell types in the spatial images above. The cell types are: **(A)** excitatory L6 neuron, **(B)** excitatory L5/6 neuron, **(C)** excitatory L5 neuron, **(D)** excitatory L4 neuron, **(E)** excitatory L2/3 neuron, **(F)** oligodendrocytes, **(G)** astrocytes, **(H)** microglia, and **(I)** inhibitory neurons. In the excitatory neurons, there is a strong pattern of layer specificity (A-E). In the non-neuronal cell types, only the oligodendrocytes exhibit a pattern of layer specificity (F-H). Inhibitory neurons do not generally show layer specificity as shown in (I).

## **Figure S3.** Venn Diagram of Risk Gene Extraction.


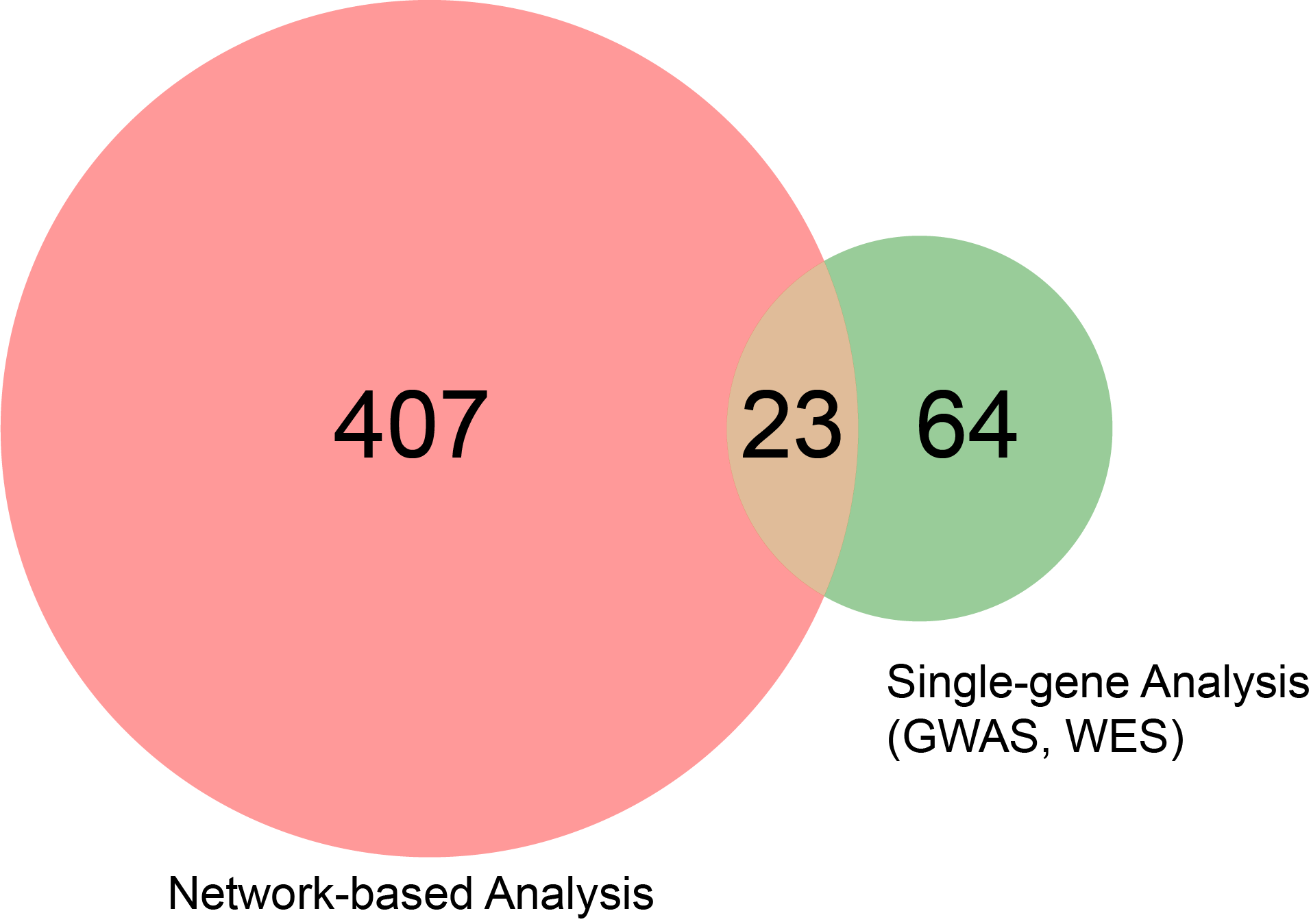


We obtained 430 genes from the network-based study. We obtained 87 risk genes from the GWAS and the exome study. We decided to include the network-based genes, in addition to the GWAS and Exome single-gene analyses, because genes do not act in isolation but in concert with others.

## **Figure S4.** Barplots of cell numbers and ligand-receptor interaction counts.


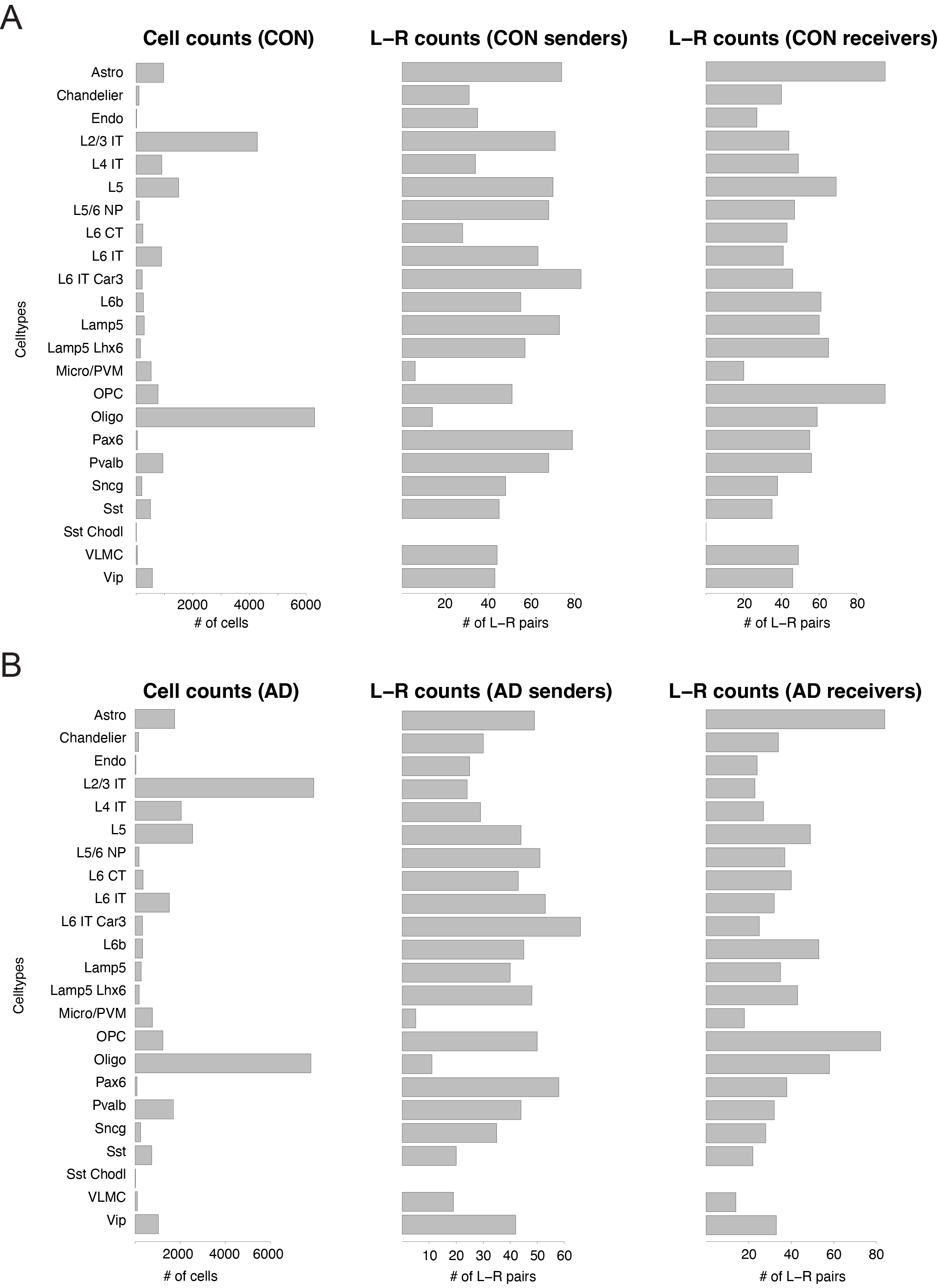


We show the number of cells and the ligand-receptor interactions for the **(A)** Control and **(B)** Alzheimer’s Disease’s C2C communication networks. From visual inspection, we see that the number of interactions is not biased based on cell counts.

## **Figure S5.** Pattern Analysis of the Incoming Cell-to-Cell Communication.


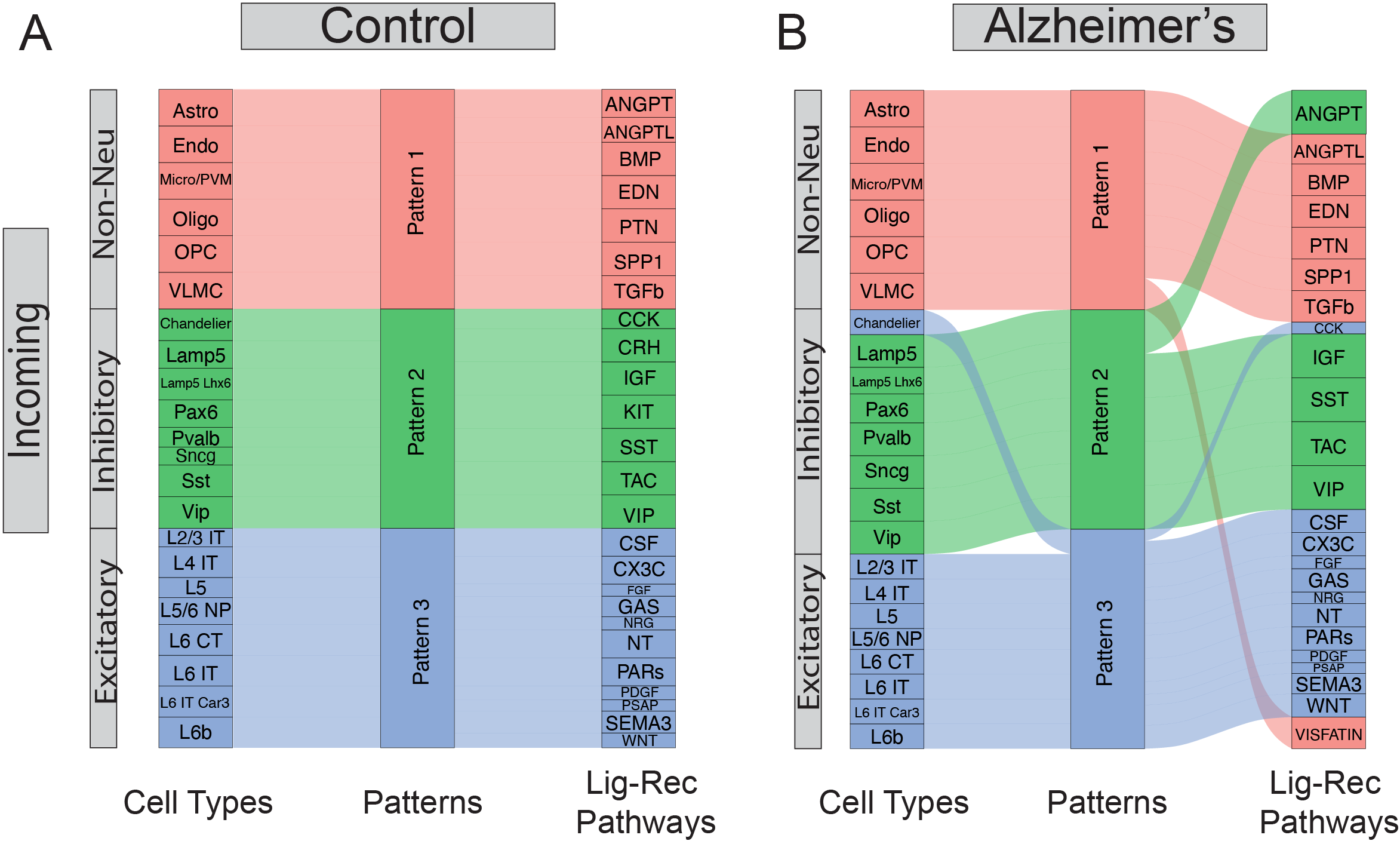


**(A)** Pattern analysis of the incoming network for the control cells. **(B)** Pattern analysis of the incoming network for the Alzheimer’s cells. The first column represents the cell types, while the last column represents the ligand-receptor pathways. Each pathway can contain multiple ligand-receptor pairs.

## **Figure S6.** Organization of our GitHub Repository.


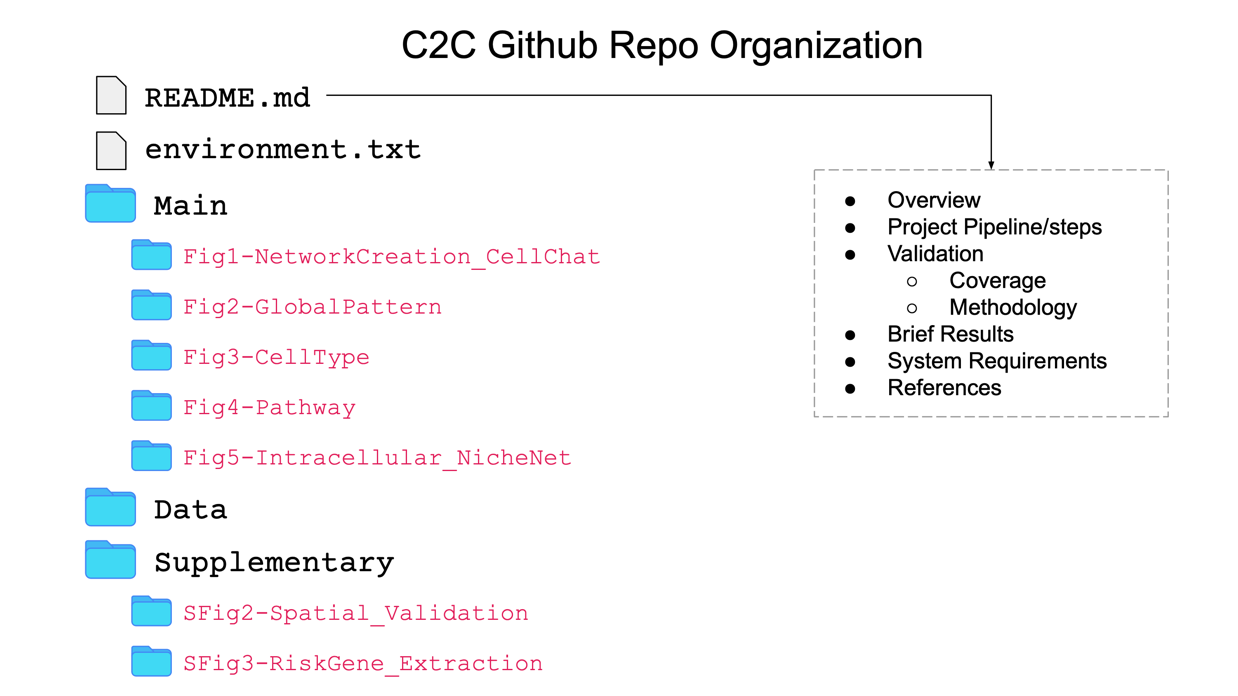


We have made our C2C computational pipeline publicly available on the GitHub repository: <https://github.com/dssikdar/C2Cv0.git>. Our code reflects our manuscript, organized by each main figure’s analysis. We also include how we extracted risk genes and performed spatial validation of our communication networks.

# Supplementary Tables

## **Table S1.** Aggregated Communication for the Outgoing Network.


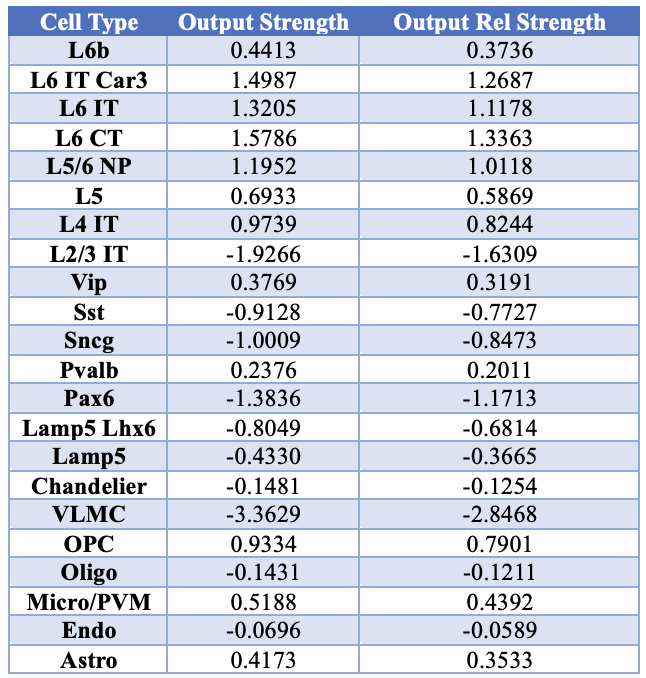


These are the aggregated output communication values for each of the sub-cell-types. The last column `Output Rel Strength` is the z-score of each entry relative to the other cell types. The mean and standard deviation of `Output Strength` were 0 and 1.1813, respectively.

## **Table S2.** Distribution of Communication for the Outgoing Network.


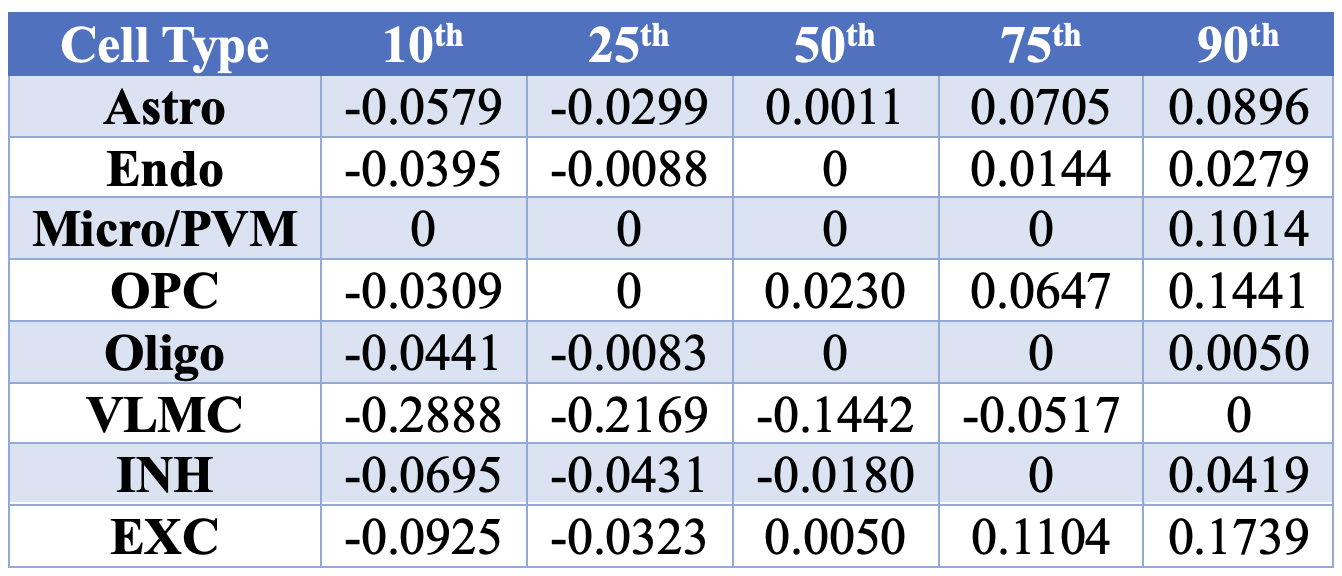


These are the percentile values for the output communication values for each of the cell types.

## **Table S3.** Aggregated Communication for the Incoming Network.


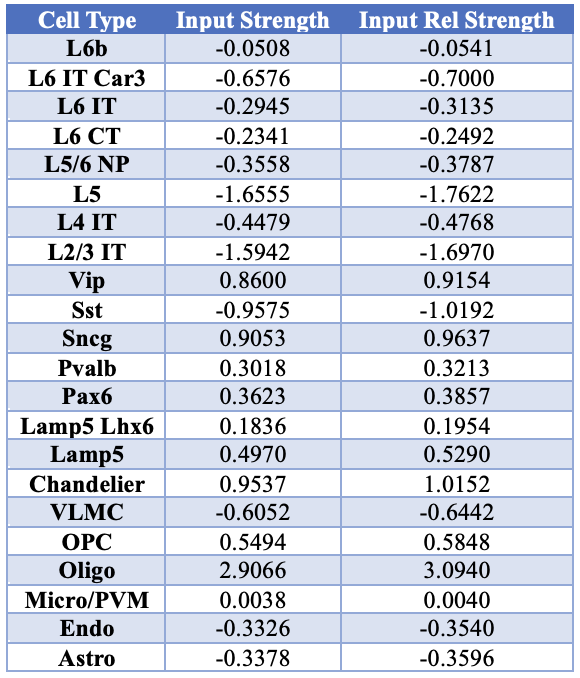


These are the aggregated input communication values for each of the sub-cell-types. The last column `Input Rel Strength` is the z-score of each entry relative to the other cell types. The mean and standard deviation of `Input Strength` were 0 and 0.9394, respectively.

## **Table S4.** Distribution of Communication for the Incoming Network.


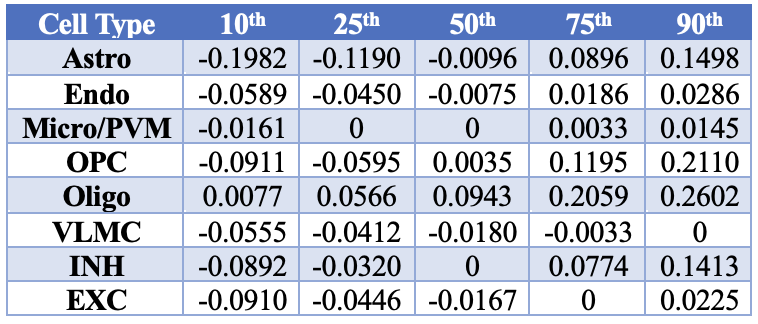


These are the percentile values for the input communication values for each of the cell types.

## **Table S5.** Ratio and P-values of each Communication Pathway.


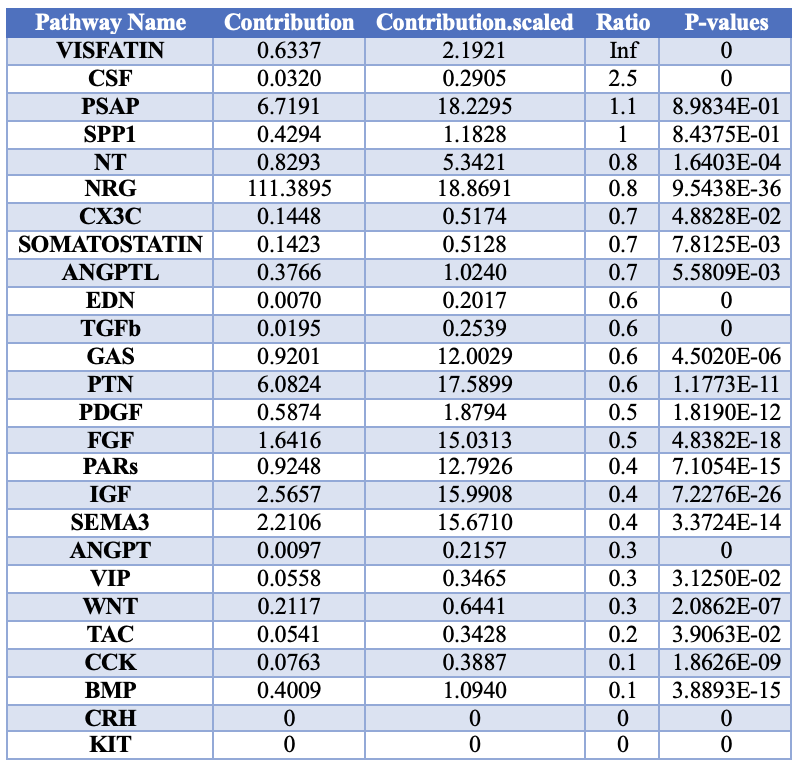


This is the result of CellChat’s paired sample Wilcoxon test comparing all possible sender-receiver cell type combinations between AD and CON groups.

## **Table S6.** Alzheimer’s Risk Genes.


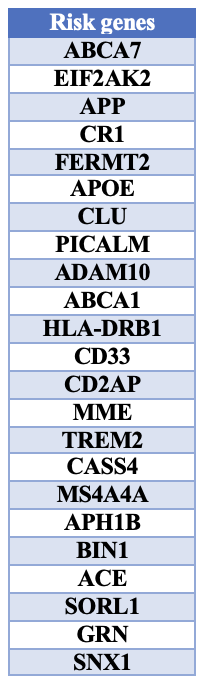


These are the 23 intersected risk genes. Risk gene extraction is described in **Methods 2.4**.

## **Table S7.** Cell and Ligand-Receptor Count Correlation.


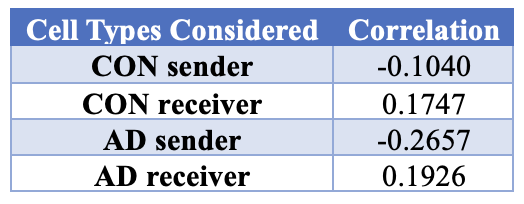


We performed Pearson correlations between the number of cells and the number of ligand-receptor interactions. Since a ligand-receptor interaction involves both a sender cell and a receiver cell, each network (i.e. CON and AD) have 2 correlation values. We found that there were low correlations between the number of cells and the number of ligand-receptor interactions.

# Supplementary Methods

## **Methods S1.** QC and Preprocessing.

Doublet Detection:

Doublets were identified using a combination of two computational methods Scrublet and DoubletDetection. The intersection of high-quality cells was taken from both software. The decision to utilize each software was based on our empirical testing and experience. For example, on our dataset, by running Scrublet and DoubletDetection sequentially, we detected an union of 10,607 doublets. For running them separately, Scrublet detected 283 doublets, DoubletDetection detected 10,475. The intersection has only 151 doublets, which shows the value of running both software.

Quality Control (QC):

For each sample, quality control metrics were assessed to ensure the integrity of the data. We filtered cells based on mitochondrial gene expression, gene count, and UMI count thresholds to remove low-quality cells. Specifically, cells with more than 10% mitochondrial gene expression, fewer than 200 genes, or fewer than 500 UMIs were excluded from further analysis.

*pg.qc_metrics(data, mito_prefix='Mito-', percent_mito=10, min_genes=200, min_umis=500)*

*pg.filter_data(data)*

We also filtered out mitochondrial, ribosomal, and sex-linked genes to minimize technical variations that might obscure biological signal utilizing packages from CellBender and MitoCarta.

Normalization and Variable Feature:

The data were then log-normalized to stabilize the variance across the dataset. We identified and retained robust genes across cells to focus on the most informative features for downstream analysis.

*pg.highly_variable_features(data, n_top=2000)*

Dimensionality Reduction and Batch Correction:

Principal component analysis (PCA) was used to reduce the dimensionality of the dataset, and Harmony was applied for batch correction to align cells across different samples, ensuring that subsequent analyses are not confounded by batch effects.

*pg.pca(data, n_components=14)*

*pca_key = "pca"*

Nearest Neighbors, Clustering, and UMAP:

We constructed a nearest-neighbor graph and performed Leiden clustering at a resolution of 4.0 to identify distinct cell populations. Uniform Manifold Approximation and Projection (UMAP) was then employed for visualizing the high-dimensional data in two dimensions, which aids in identifying and interpreting cell clusters.

*pg.neighbors(data, rep=pca_key, n_jobs=15)*

*pg.leiden(data, resolution=4, rep=pca_key)*

*pg.umap(data, rep=pca_key, n_jobs=15)*

Refinement of Clusters:

Post initial clustering, we evaluated the cluster integrity by checking for overrepresentation of cell types within clusters. Clusters expressing multiple cell types or having low gene counts were pruned to refine the population structure.

Iterative Analysis:

We iteratively performed HVG selection, PCA, batch correction, and clustering, followed by UMAP visualization. This iterative process was repeated until the data quality and cluster resolution met our stringent criteria for robust cell type identification.

These preprocessing steps were critical in ensuring that our final dataset was of high quality and suitable for downstream analysis, including the examination of differential ligand/receptor expression, cell type annotation, and elucidation of cell-cell communication.
